# Non-invasive applications of Raman spectroscopy in assisted reproduction

**DOI:** 10.3389/fendo.2025.1577702

**Published:** 2025-05-08

**Authors:** Mingxing Sui, Lihui Si, Zhuoyue Chen, Yingli Lu, Hongru Li

**Affiliations:** ^1^ Department of Obstetrics and Gynecology, The Second Hospital of Jilin University, Changchun, Jilin, China; ^2^ College of Biological and Agricultural Engineering, Jilin University, Changchun, Jilin, China

**Keywords:** Raman spectroscopy, assisted reproductive technology (ART), gamete quality, embryo viability, non-invasive assessment

## Abstract

This review explores the non-invasive applications of Raman spectroscopy in assisted reproductive technology (ART). Raman spectroscopy, a powerful tool for analyzing biological samples, has shown great potential in enhancing ART outcomes through various applications such as sperm quality assessment, oocyte evaluation, and embryo selection. The non-destructive nature and high specificity of this technique enable detailed biochemical analysis at the cellular level, offering valuable insights into cellular processes without harming for the samples. The review highlights recent advancements and studies demonstrating the efficacy of Raman spectroscopy in improving the selection criteria for gametes and embryos, ultimately contributing to higher success rates in ART. Future perspectives on integrating Raman spectroscopy with other technologies to further enhance its applicability in reproductive medicine are also discussed.

## 1 Introduction

### 1.1 Infertility and the role of ART

Infertility is a significant global health concern, impacting approximately 15% of individuals of reproductive age. Assisted reproductive technology (ART) represents the most effective treatment option for infertile couples. In ART procedures like *in vitro* fertilization-embryo transfer (IVF-ET), sperm and oocyte are handled and fertilized *in vitro*. The highest-quality embryo is then selected for transfer. Assessing the quality of gametes and pre-transfer embryos is of utmost importance.

### 1.2 Definition and principles of Raman spectroscopy

**Figure 1:**
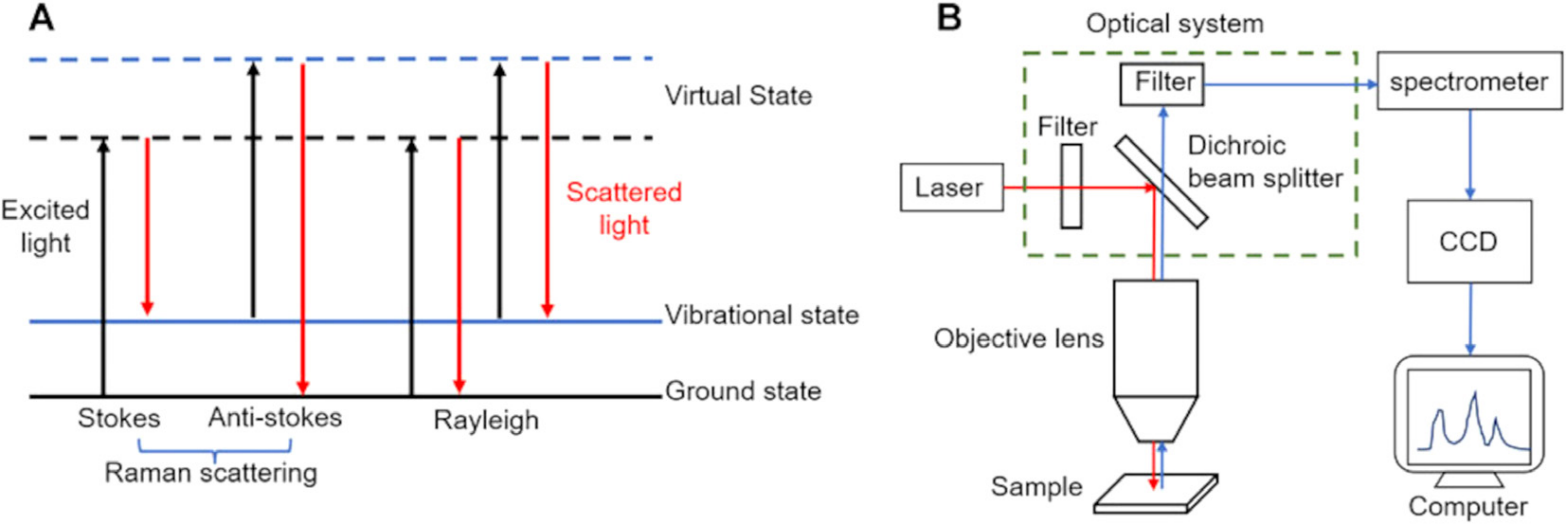
Principles of Raman spectroscopy. **(A)** process of light absorption and scattering in Raman spectroscopy; **(B)** components and setup of a typical Raman spectrometer. Figures modified from Ref. (2).

Raman spectroscopy is based on the principle of light scattering, where photons interact with the molecules of a substance, causing the light to scatter. When a monochromatic laser beam illuminates a sample, most of the light is elastically scattered (Rayleigh scattering), retaining the same energy as the incident photons. However, a small fraction of the light undergoes inelastic scattering (Raman scattering) (1), where the energy of the scattered photons changes due to interactions with molecular vibrational or rotational energy levels. These energy shifts provide detailed molecular information (**Figure 1**) (2). Each molecule has unique vibrational modes, producing specific energy changes observable as shifts in the Raman scattered light. These shifts are represented in a Raman spectrum, where the horizontal axis indicates the Raman shift (in wavenumbers, cm^-1^) and the vertical axis shows the intensity of the scattered light. The Raman shift corresponds to the difference in energy between the incident and scattered photons, reflecting the molecular vibrational frequencies.

Mathematically, the energy of the incident photon ***E_i_*** = **hv*_i_*** and the scattered photon can be expressed as **E**_s_ = **h**(**v**_i_−v_**m**_) for Stokes scattering (energy decrease) and **E**_**s**_ = **h**(v_**i**_+v_**m**_)for anti-Stokes scattering (energy increase), where is the molecular vibrational frequency. The Raman shift s thus given by **v**_**i**_−**v**_**s**_ or Stokes and **v**_**s**_−**v**_**i**_ for anti-Stokes scattering. These unique shifts form a molecular “fingerprint” in the Raman spectrum, enabling the identification and analysis of different molecular components and structures. This non-destructive, label-free technique provides high spatial and chemical resolution, making it an invaluable tool for various applications, including live cell monitoring, material science, and paleobiological studies. Currently, several primary types of Raman spectroscopy are employed, encompassing resonance Raman spectroscopy, coherent anti-Stokes Raman scattering (CARS), stimulated Raman scattering (SRS) microscopy, and surface-enhanced Raman scattering (SERS) (3). Additionally, emerging techniques such as laser tweezers Raman spectroscopy (LTRS) and micro-Raman spectroscopy are also notable in the field.

### 1.3 Brief history and development of Raman spectroscopy

Raman spectroscopy, discovered by Sir C.V. Raman in 1928 and honored with the Nobel Prize in Physics in 1930, has evolved into a powerful tool for non-invasive, label-free monitoring of cellular processes, thus providing a detailed molecular fingerprint of cell biochemical composition (4). Early challenges, such as the weak signal compared to fluorescence, spurred significant progress in laser technology and detector sensitivity over subsequent decades (5). Key milestones include the introduction of Fourier Transform Raman spectroscopy in the 1970s, a technique which revolutionized spectral resolution and sensitivity, enabling precise analysis of complex molecular structures (6). The integration of Raman spectroscopy with microscopy techniques in the 1980s allowed for spatially resolved analysis at the microscopic level, profoundly impacting biological and materials science research. Advances in detectors and imaging technologies further enhanced Raman spectroscopy capabilities, with Charge-Coupled Device (CCD) detectors and confocal Raman microscopy enabling high-resolution imaging and molecular mapping (7). The development of SRS in the 1990s marked a significant breakthrough, offering enhanced sensitivity and faster imaging capabilities, which proved particularly valuable in biological studies and real-time monitoring of living cells (8). Around the same time, CARS also gained attention as a powerful nonlinear optical method for rapid imaging of molecular vibrations, particularly useful in tissue and live-cell imaging. These developments collectively transformed Raman spectroscopy from a laboratory curiosity into a sophisticated analytical tool used across scientific disciplines, including chemistry, materials science, biology, and medicine, driving continuous innovation in the field. Unlike traditional fluorescence imaging, Raman imaging maps chemical bonds in unlabeled samples, allowing analysis of biomolecular heterogeneity and disease phenotypes (9). Raman spectroscopy maintains cell viability and enables post-analysis clinical applications.

### 1.4 Importance of Raman spectroscopy in biomedical research

Raman spectroscopy is profoundly important in biomedical research due to its capacity for non-destructive, label-free analysis of biological samples, offering detailed molecular insights into tissues, cells, and biofluids (10). This technique enables the characterization of key biomolecules like proteins, lipids, nucleic acids, and carbohydrates, providing crucial information about cellular structure and composition. For instance, in cancer research, Raman spectroscopy has been used to differentiate between healthy and malignant tissues based on molecular signatures, aiding in early diagnosis and guiding surgical interventions and prognostic assessment, for example, distinguish between skin cancer (11), cervical cancer (12), breast cancer (13), lung cancer (14), gastric cancer (15), brain cancer (16), laryngeal cancer (17), bladder cancer (18), kidney cancer (19). Additionally, in neurological disorders, Raman spectroscopy has helped identify biochemical changes associated with diseases like Alzheimer’s and Parkinson’s, revealing potential diagnostic biomarkers. In drug development, Raman spectroscopy plays a pivotal role in studying drug-target interactions, assessing drug delivery systems, and monitoring drug distribution within tissues. For example, researchers use Raman spectroscopy to analyze cellular response to drug treatments, facilitating the development of personalized therapies. Moreover, in tissue engineering and regenerative medicine, Raman spectroscopy contributes to optimizing cell culture conditions, monitoring tissue growth, and evaluating biomaterial integration by providing real-time, molecular-specific information. Overall, Raman spectroscopy’s ability to probe biological systems at the molecular level has transformed biomedical research, enabling a deeper understanding of disease mechanisms, informing drug development, and guiding therapeutic interventions.

## 2 Application of Raman spectroscopy in assisted reproduction

**Figure 2:**
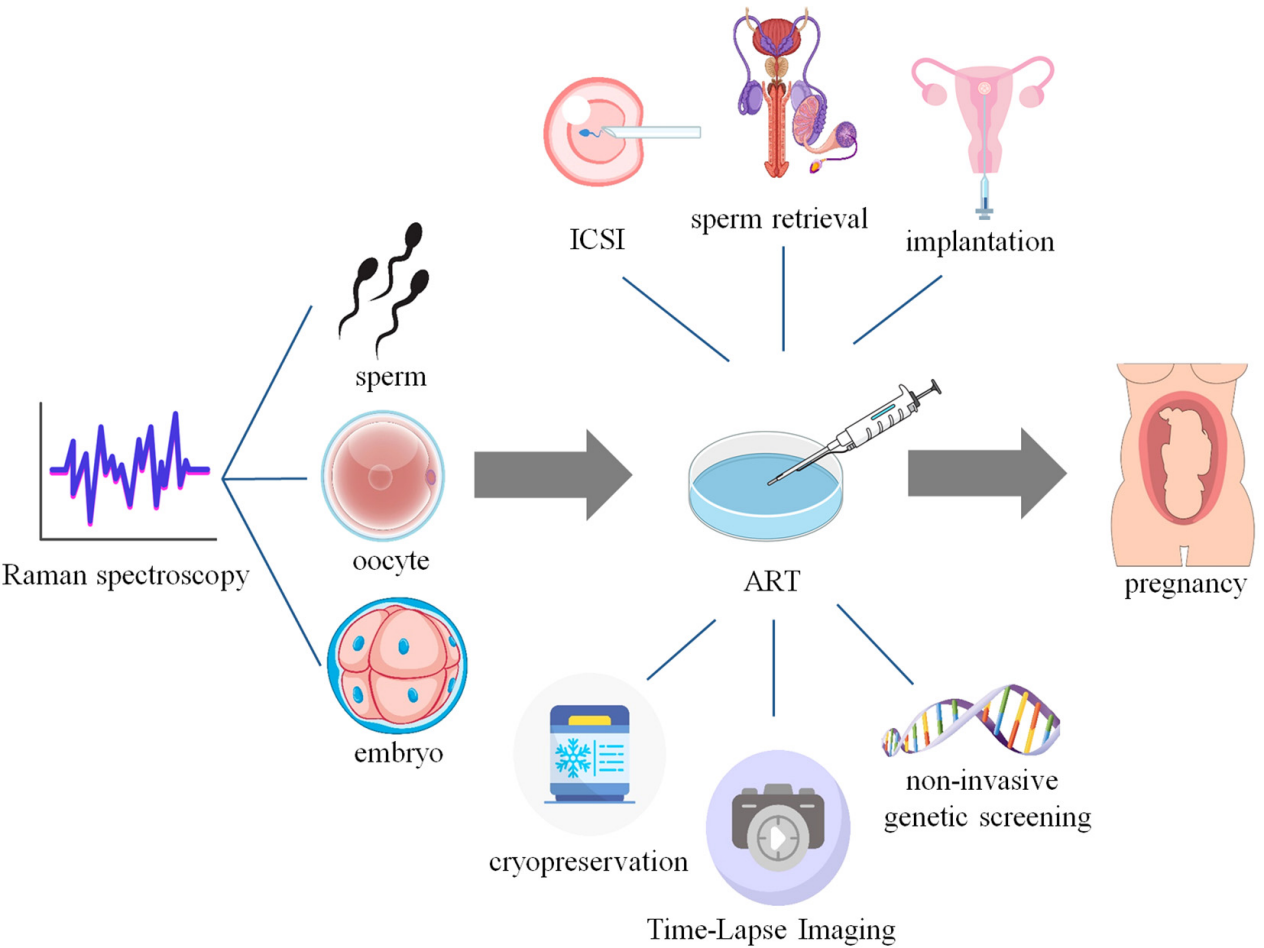
Application of Raman spectroscopy in Assisted Reproduction. This figure created with elements in website Vecteezy and Scidraw.

With the increasing demand for more precise and non-invasive evaluation methods in ART, Raman spectroscopy has emerged as a transformative analytical technique in the field of assisted reproduction, which provides unprecedented insights into the molecular and biochemical properties of reproductive cells and tissues. The aim of this review is to highlight the utility and potential of Raman spectroscopy in assisted reproduction to enhance reproductive outcomes (**Figure 2**).

### 2.1 Sperm

Raman spectroscopy has emerged as a pivotal analytical tool in male assisted reproduction, offering unique insights into sperm function and fertility through non-invasive, label-free analysis of molecular and biochemical aspects (20). This technique provides detailed information about the molecular composition of cells, enabling quantification of biomolecular constituents such as nucleic acids, phospholipids, and structural proteins in single sperm cells (21), making it suitable for sensitive samples like sperm, and has shown potential in evaluating chromatin condensation and distinguishing between X and Y chromosome-bearing sperm, therefore supporting spermatogenesis and fertility treatments. Studying sperm epigenetic markers is crucial for understanding male idiopathic infertility (22). Changes in DNA packaging or epigenetic modifications can be important (23, 24), but these small details are difficult to evaluate by standard methods without disturbing or even destroying the sperm cell (25). Standard sperm sorting approaches primarily rely on visible traits such as motility and form (26–28), but without addressing sensitivity to deeper genomic or epigenetic abnormalities (29–31). Therefore, alternative methods like microfluidics, nanopurification, and Raman spectroscopy, which can isolate sperm based on novel parameters without harming them, allowed principal component analysis (PCA) and discriminant function analysis (DFA) to be performed on fresh, stained, sorted and frozen-thawed sperm, are promising for monitoring sperm function and quality (32).

#### 2.1.1 Sperm quality assessment

##### 2.1.1.1 mitochondrial function

One of the significant applications of Raman spectroscopy in this field is the study of mitochondrial function in sperm cells. Abramczyk et al. (33) demonstrated the utility of Raman imaging in assessing the redox state of cytochrome c within sperm mitochondria. The findings indicated that the redox balance of cytochrome c plays a crucial role in sperm motility and fertilization capability, highlighting the dual functionality of cytochrome c in both energy production and apoptotic processes. This study underscores the importance of mitochondrial dynamics in sperm physiology and provides a foundation for further exploration into the metabolic disorders that could impair male fertility. Specifically, the research involved obtaining semen samples from 11 donors after 2–7 days of sexual abstinence, consistent with WHO guidelines for semen analysis. Subsequent Raman imaging of these samples allowed for the analysis of specific biochemical components within sperm cells, revealing characteristic peaks (750, 1127, 1311, 1399, and 1582 cm^-1^) associated with cytochrome c in mitochondria. Through PCA analysis, the study successfully differentiated sperm cell regions (head, midpiece, and tail) based on their biochemical profiles, particularly emphasizing the redox-balanced forms of cytochrome c in the midpiece region. The analysis highlighted varying intensities of cytochrome c Raman bands across different sperm cells, suggesting potential differences in mitochondrial function and redox status among donors. These findings provide valuable insights into mitochondrial dynamics and their implications for sperm quality and male fertility, advancing our understanding of molecular mechanisms underlying male reproductive cell function and dysfunction pioneered by Kubasek et al. (34) in 1986.

##### 2.1.1.2 Sperm nuclei and DNA integrity

**Figure 3:**
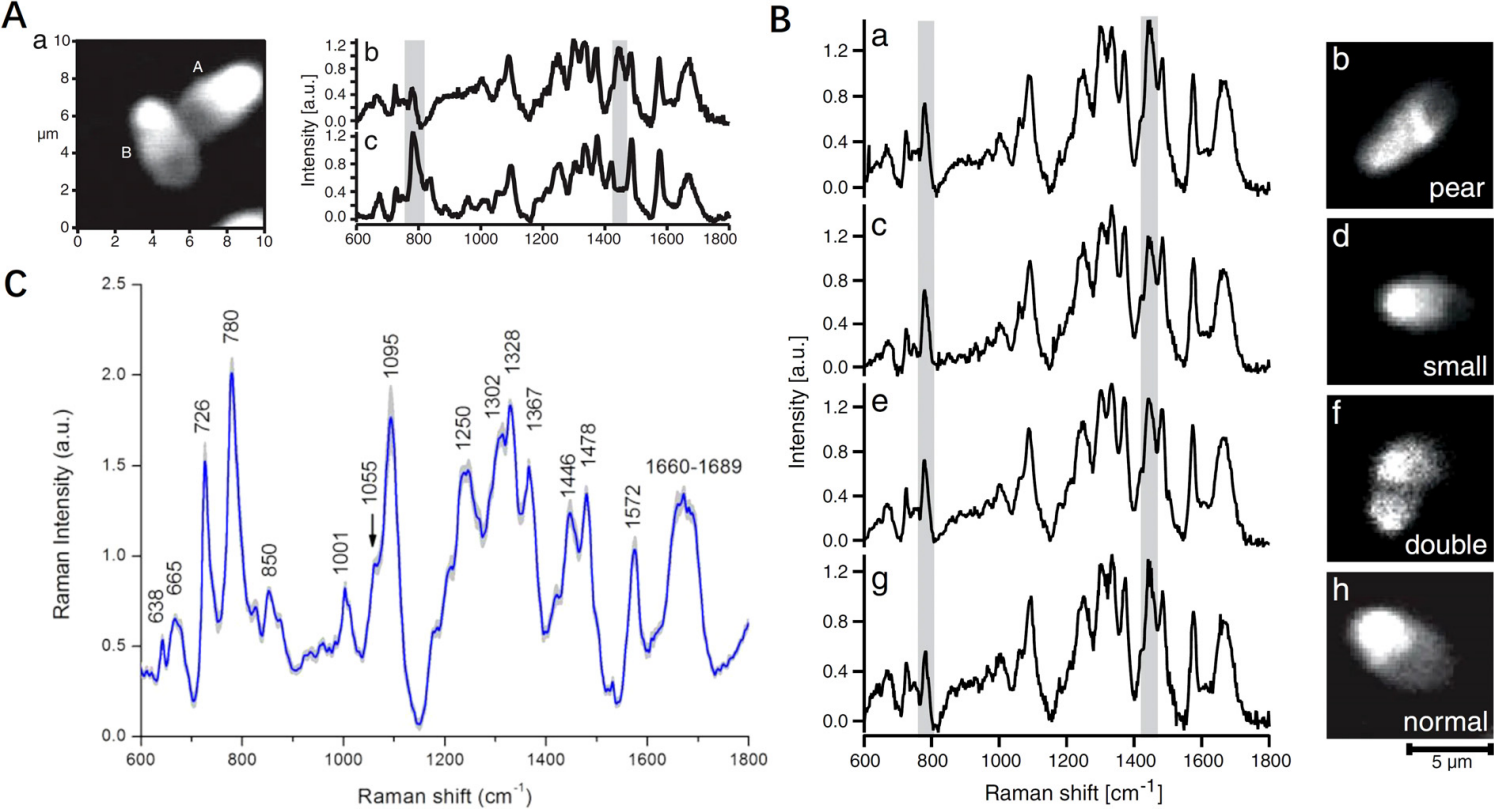
Comparison of morphology and biochemical composition of human sperm cells using Raman spectroscopy. **(A)** Spontaneous micro-Raman spectrum of the central part of normal sperm head and calf-thymus DNA gel; **(B)** peak intensities relative to DNA backbone vibration for differentiating pear, small, double and normal sperm cell shapes; **(C)** intensity ratio of Raman bands (1055/1095 cm^-1^) indicating a potential biomarker for assessing the human sperm DNA integrity. **(A, B)** modified from Ref. (35). **(C)** modified from Ref. (38).

Several studies have effectively applied Raman spectral profiling to evaluate nuclear architecture and chromatin packaging efficiency of spermatozoa, highlighting its potential in reproductive health diagnostics and assisted reproduction technologies, especially in sperm selecting and intracytoplasmic sperm injection (ICSI). Huser et al. (35) utilized micro-Raman spectroscopy to analyze DNA packaging in individual human sperm cells, successfully differentiating normal from abnormal cells by assessing DNA-protein complex structures and packaging efficiency. Notably, the intensity of the 785 cm^-1^ band serves as a marker for the efficiency of DNA packaging, with a low intensity indicating highly efficient protein binding (**Figure 3A**). The variations in the intensity ratio of 785 cm^-1^/1092 cm^-1^ and 1442 cm^-1^/1092 cm^-1^ were used to discriminate between normal and abnormal sperm cells (**Figure 3B**). This significant discovery was followed by substantial progress in the application of Raman spectroscopy to differentiated normal and abnormal semen samples made by Huang’s team (36–38). The authors found that the two could be clearly differentiated based on the peak ratios of 1,449 cm^-1^ to 1,418 cm^-1^ (36). Subsequently, his team discovered that lefthanded polarized SERS spectroscopy yielded the best diagnostic results, with a sensitivity of 95.8% and specificity of 64.9% (37). Furthermore, they proposed that the intensity ratio between 1,055 cm^-1^ and 1,095 cm^-1^ from the obtained Raman spectra, combined with image analysis, could serve as a potential biomarker for assessing sperm DNA integrity (**Figure 3C**) (38). Meanwhile, Li et al. (39) showed that melatonin, analyzed via laser tweezers Raman spectroscopy, protects buffalo sperm from reactive oxygen species, improving semen quality after freezing and thawing. Subsequently, a PDMS microfluidic platform was introduced for single sperm analysis (40), suitable for non-invasive Raman spectroscopy, allowing for viability, chromosome content, and acrosome status studies. Costa et al. (41) focused on the posterior acrosomal region, where DNA concentration is highest, using a 632.8 nm He-Ne laser, revealing detailed patterns in sperm DNA spectra. Pachetti (42) employed UV Resonance Raman spectroscopy with a synchrotron radiation source at a 250 nm wavelength to analyze RNA from spermatozoa, focusing on adenine methylation. This method overcame fluorescence interference, enhancing adenine and guanine contributions, and addressed challenges related to low RNA concentration and contamination, using UV/Vis absorbance measurements for RNA quantification and purity assessment. Additionally, Jahmani et al. (43) demonstrated that confocal Raman spectroscopy could detect chromatin condensation levels in sperm, with significant Raman peaks correlated with Chromomycin A3 staining. Du et al. (44) differentiated intact and damaged sperm DNA on glass slides using micro-Raman spectroscopy, achieving high diagnostic sensitivities and specificities through multivariate analysis, indicating distinct molecular signatures associated with DNA fragmentation. Potential biomarkers for sperm DNA integrity were identified using specific Raman peak ratios, enhancing the ability to assess sperm quality. These studies highlight the important potential of Raman spectroscopy for sperm genome quality assessment and selection of sperm for assisted fertilization, by enabling detailed molecular assessments of sperm DNA integrity and structure.

#### 2.1.2 X and Y chromosomes

Raman spectroscopy has proven valuable in studying bovine sperm, with applications in both biochemical and morphological characterization (45). Ferrara et al. (46) combined Raman spectroscopy with digital holography to investigate sperm cells, revealing higher DNA concentrations in X-bearing sperm compared to Y-bearing sperm, as indicated by specific Raman peaks (726, 785, 1095, and 1581 cm^-1^). This label-free approach complements traditional staining methods. In a related study, Li et al. (47) utilized laser confocal Raman spectroscopy to differentiate between X and Y chromosome-bearing sperm based on DNA backbone and skeleton regions. X sperm exhibited elevated peak values at 785 cm^-1^ (DNA backbone) and 1095 cm^-1^ (PO_4_ skeleton) compared to Y sperm, with significant differences in Raman spectral features confirmed through statistical analysis. This non-invasive technique shows promise in predicting sperm aneuploidy, particularly relevant for improving outcomes in ART like preimplantation genetic testing for aneuploidies (PGT-A). By aiding in the selection of embryos with balanced chromosomal content, Raman spectroscopy contributes to enhancing the success of fertility treatments and genetic screening processes.

#### 2.1.3 Spermatogenesis and surgical sperm retrieval

**Figure 4:**
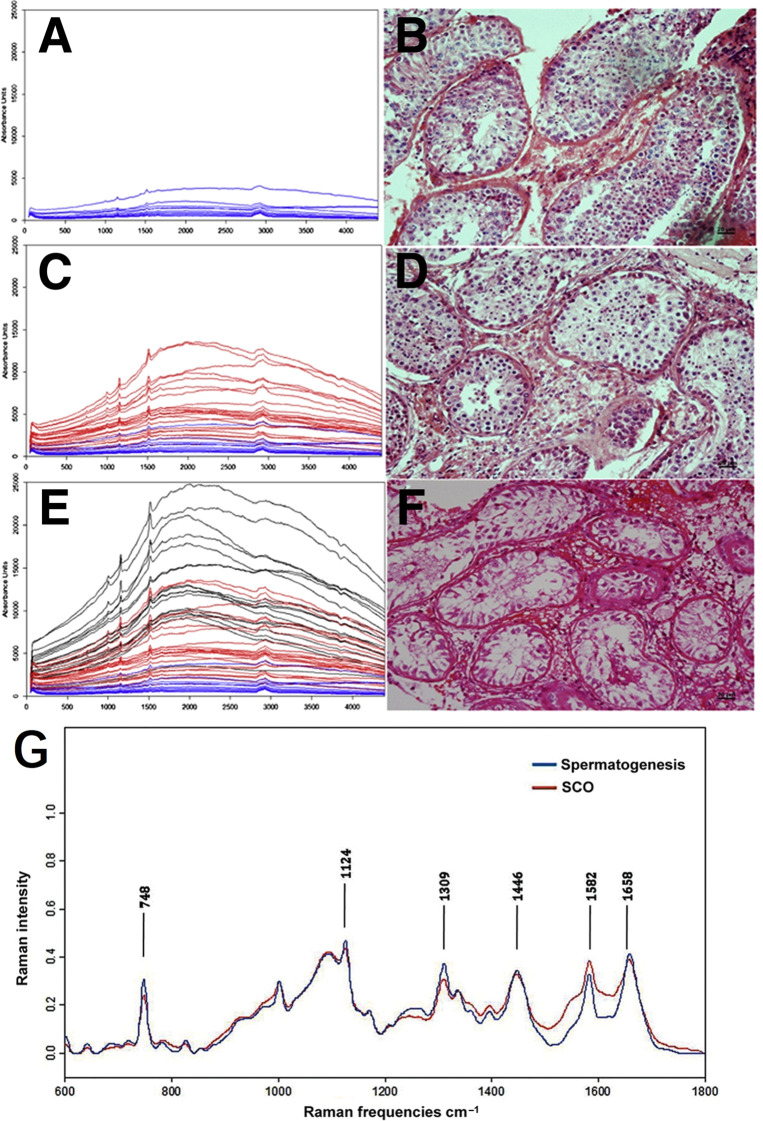
Raman spectroscopy in distinguishing between complete and incomplete spermatogenesis within human seminiferous tubules. **(A, C, E)** Raman spectra of seminiferous tubules at various stages of maturation; **(B, D, F)** corresponding H&E histological diagnosis; **(A, B)** tubules with spermatogenesis; **(C, D)** MA tubles; **(E, F)** SCA tubles; **(G)** normalized average Raman spectra of tubules with spermatogenesis and SCO tubules. Figures **(A-F)** modified from Ref. (50). **(G)** modified from Ref. (51).

In male infertility treatment, sperm retrieval from non-obstructive azoospermia (NOA) patients, where sperm are scarce or absent, typically involves microdissection testicular sperm extraction (microTESE). Despite its improvement over traditional biopsy methods, microTESE remains inefficient, costly, and time-consuming (48). Raman spectroscopy offers detailed molecular insights and has been effective in differentiating spermatogenic activity within testicular tissues and assessing sperm DNA integrity, critical for successful ART (49). Liu (50) employed Raman spectroscopy to differentiate between complete and incomplete spermatogenesis in human seminiferous tubules, focusing on patients with obstructive azoospermia (OA) and NOA. Raman intensities for each group were approximately >8000 (au) for Sertoli cell-only (SCO) tubules, 2000–10000 (au) for maturational arrest (MA) tubules, and <2000 (au) for tubules with spermatogenesis. The study revealed that NOA tubules exhibited significantly higher intensities at 1001 cm^-1^, 1152 cm^-1^, 1515 cm^-1^, and 1658 cm^-1^ compared to OA tubules, with these peaks corresponding to proteins, suggesting increased protein content in NOA tubules (**Figures 4A-F**). Raman spectroscopy showed high sensitivity (90%) and specificity (85.71%) in detecting the presence of sperm, making it a valuable tool for guiding micro-testicular sperm extraction procedures. This noninvasive technique represents a significant advancement in male infertility treatment, with the potential to improve sperm retrieval rates. His team further evaluated Raman spectroscopy’s ability to distinguish tubules with spermatogenesis, revealing that spectra of tubules with spermatogenesis exhibited stronger peaks at 748, 1124, 1309, 1446, and 1658 cm^-1^ compared to SCO tubules, with a notable decrease observed at 1582 cm^-1^, achieving a sensitivity of 91.2% and specificity of 82.9% (51) (**Figure 4G**). Ma et al. (52) also applied Raman spectroscopy to distinguish Sertoli cells in testes of patients with obstructive and NOA. Unlike traditional testicular biopsy, Raman spectroscopy provides a non-invasive way to evaluate spermatogenesis without the need for external labeling agents. This technology is promising for identifying seminiferous tubules with active spermatogenesis during micro-TESE procedures, potentially enhancing sperm retrieval rates through the concept of Raman-guided micro-TESE, which can lead to better fertility treatment outcomes.

### 2.2 Oocyte

In female reproductive physiology, oocytes are crucial for reproduction as they are the precursor cells to embryos upon fertilization. Oocytes develop within ovarian follicles, fluid-filled structures in the ovaries that provide essential nutrients and signaling molecules necessary for oocyte maturation and successful fertilization. Raman spectroscopy is used for assessing the molecular composition and quality of oocytes and follicular fluid in ART. This technique offers detailed insights into the biochemical environment of oocytes and the surrounding follicular fluid without causing damage to these delicate reproductive cells, and enhances our understanding of oocyte quality and assists in optimizing ART outcomes. The ability to analyze the biochemical environment of oocytes non-invasively underscores the importance of Raman spectroscopy in personalized reproductive healthcare, where tailored approaches based on molecular profiling contribute to improved fertility treatment efficacy.

#### 2.2.1 Oocyte quality assessment

The quality of oocytes is crucial for the developmental potential of embryos post-fertilization (53), but the traditional non-invasive selection method based on morphological criteria is highly subjective and does not consistently reflect the true developmental capacity of the oocytes (54). By capturing metabolic, structural, and subcellular dynamics, Raman spectroscopy provides a comprehensive means to assess oocyte quality and identify biomarkers indicative of developmental potential.

**Figure 5:**
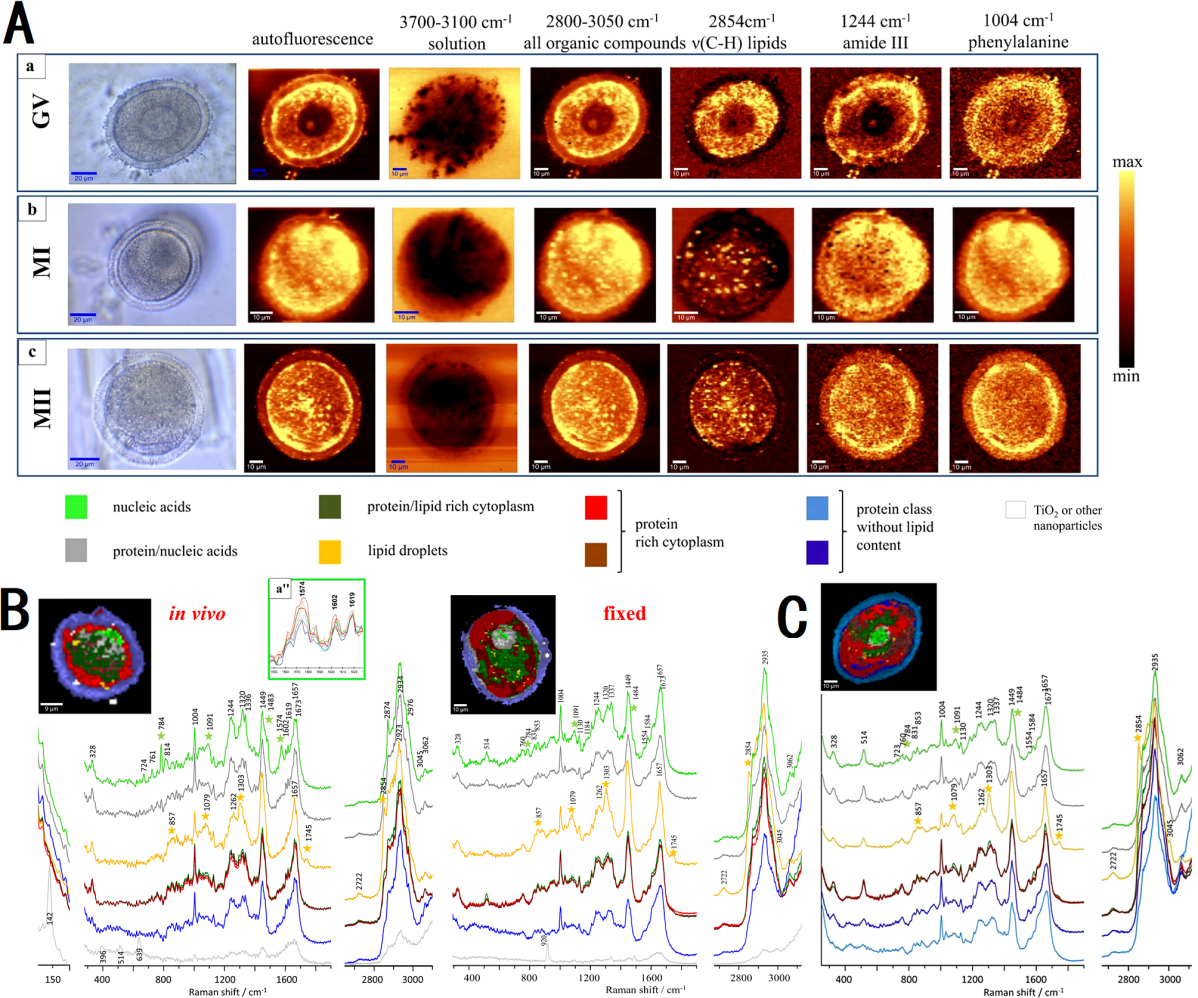
Micrographs showing the intensity of Raman spectral bands related to oocyte composition of mouse oocytes at various maturation stage. **(A)** micrographs through the central plane of live oocytes at GV, MI and MII stage; **(B)** comparison between functional and fixed MII oocytes; **(C)** micrographs through two horizontal image planes of fixed oocytes at MI stage. Figures modified from Ref. (55).

Heraud et al. (55) utilized confocal Raman microspectroscopy to perform detailed three-dimensional imaging and composition analysis of live murine oocytes at various maturation stages (GV, MI, MII, **Figure 5A**). Their study highlighted significant alterations in macromolecular chemistry due to fixation (**Figures 5B, C**), identifying a specific mitochondrial function marker band at 1602 cm^-1^ present only in live oocytes. The imaging techniques revealed dynamic changes in nuclear organization and cytoplasmic architecture, such as chromatin condensation, mitochondria aggregation, and lipid droplet variations. These findings underscore the importance of three-dimensional imaging for accurately assessing the structure and functionality of oocytes.

**Figure 6:**
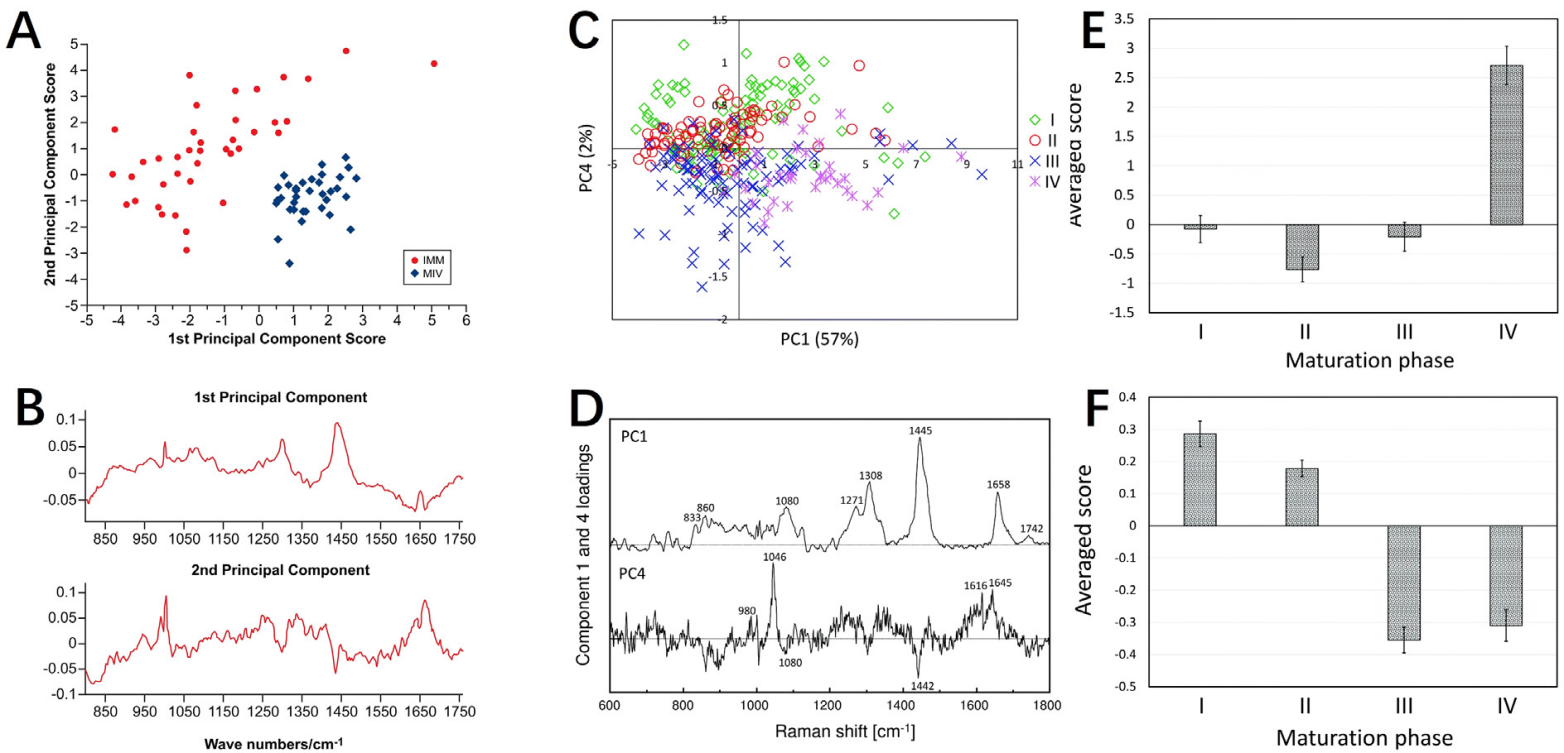
Raman spectroscopy for assessing oocyte maturation status. **(A, B)** PCA results and Raman spectrograms of immature oocytes and *in vitro* matured mouse oocytes; **(C–F)** PCA results and Raman spectrograms of mouse oocytes at different maturation stages (I, II, III, IV). **(A, B)** modified from Ref. (56). **(C–F)** modified from Ref. (57).

It is the exploration of the biochemical composition and developmental stages of oocytes that the application of Raman spectroscopy in ART still focuses on nowadays. Raman spectral analysis combined with multivariate algorithms revealed maturation-dependent biochemical patterns (56), distinguishing immature and matured oocytes through lipid- and protein-associated principal components (**Figures 6A, B**). In contrast, another research focused on *in vivo* maturation phases. PCA was performed on 324 Raman spectra of oocytes at various maturation stages, revealing distinct molecular characteristics (57). The score plots using PC1 and PC4 show a clear separation of maturation phases. PC1 distinguishes Phase IV, which has higher lipid concentrations, indicated by characteristic peaks at 1064, 1080, 1120, 1271, 1308, 1445, 1658, and 1742 cm^-1^. PC4 differentiates early (Phases I and II) from late (Phases III and IV) maturation stages, with a significant peak at 1046 cm^-1^ attributed to PO_4_^3-^ symmetric stretching, suggesting higher phosphoric acid concentrations in the early phases (**Figures 6C-F**). Meanwhile, the authors noted that Phase II oocytes exhibited higher phosphoric acid concentrations and increased protein phosphorylation compared to phases III and IV. Their study employed PCA-linear discriminant analysis (LDA) to classify Phase II oocytes with 90.7% accuracy. Both studies underscored Raman spectroscopy’s non-invasive capability to assess oocyte quality and maturation status, thereby elucidating the molecular factors influencing developmental competence across different stages.

In another investigation, the researchers demonstrated effectiveness of Raman spectroscopy in detecting oxidative damage associated with aging in mouse oocytes (58). By comparing spectra from various oocyte conditions, such as young, *in vitro* aged, oxidative-stressed, and old, the research identified significant biochemical differences, particularly in lipid and protein components. PCA highlighted distinct spectral markers indicative of oxidative damage, suggesting Raman spectroscopy as a promising non-invasive tool for assessing oocyte quality. Recently, Raman spectroscopy was utilized by Huang et al. (59) to analyze metabolic compositions in the follicular fluid of polycystic ovary syndrome (PCOS) patients. Their findings linked specific Raman biomarkers, notably peaks at 998 cm^-1^ and 1167 cm^-1^, with variations in oocyte developmental potential and clinical pregnancy rates. This approach underscores Raman spectroscopy’s potential for evaluating oocyte quality and its relevance in clinical assessments related to reproductive health.

#### 2.2.2 Oocyte vitrification

Several studies have employed Raman microspectroscopy to investigate biochemical changes in oocytes following vitrification. Bogliolo et al. (60) investigated the molecular composition of the zona pellucida (ZP) in vitrified/warmed *in vitro* matured ovine oocytes using Raman microspectroscopy. They found increased β-sheet content and decreased α-helix content within ZP proteins post-cryopreservation, underscoring significant biochemical changes induced by the process. In their following investigation (61), they utilized Raman microspectroscopy to analyze ovine oocytes vitrified using Cryotop devices. They observed significant spectral alterations in protein regions such as Amide I (1657 cm^-1^) and Amide III (1440 and 1300 cm^-1^), indicative of structural changes, including those in the cortical F-actin network. This was consistent with abnormal distributions observed via immunofluorescence imaging of F-actin, highlighting Raman microspectroscopy’s utility in assessing cytoskeletal modifications during cryopreservation. Rusciano et al. (62) employed Raman microscopy to analyze biochemical alterations in bovine oocytes post-vitrification, revealing changes in protein secondary structures and lipid configurations, which correlated with reduced developmental competence. These studies collectively demonstrate the effectiveness of Raman microspectroscopy in elucidating molecular transformations induced by cryopreservation in oocytes, thereby shedding light on the factors that influence oocyte quality and developmental outcomes.

### 2.3 Embryo

#### 2.3.1 Embryo quality and development potential assessment

**Figure 7:**
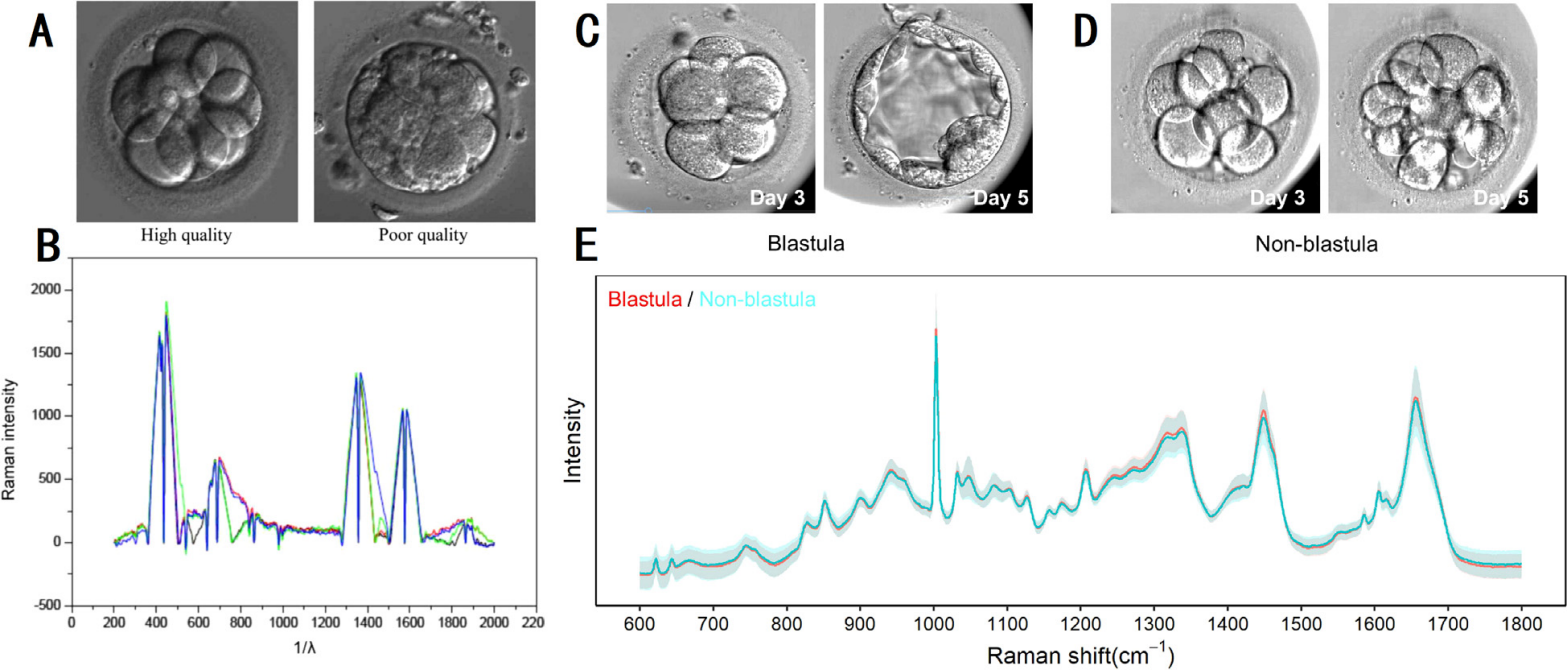
Raman spectroscopy on embryo quality and development potential assessment. **(A, B)** micrographs and Raman spectrograms of high and poor quality embryos of day 3; **(C–E)** micrographs and Raman spectrograms of embryos that progressed to the blastocyst stage and those that did not. **(A, B)** modified from Ref. (64). **(C–E)** modified from Ref. (65).

Raman spectroscopy has emerged as a valuable tool in assisted reproduction, allowing for the assessment of embryo viability and genetic characteristics through embryo culture media analysis (63). Recent studies have investigated the application of Raman spectroscopy in assessing embryo metabolism and predicting developmental outcomes during *in vitro* fertilization (IVF). Ding et al. (64) examined Raman spectroscopy’s potential by comparing its efficacy with conventional morphological scoring of day 3 embryos. Their findings revealed distinct spectral features in embryo culture media, such as peaks at 755 cm^-1^ for high-quality embryos and 750 cm^-1^ for tryptophan, indicating varying metabolic activities associated with embryo quality (**Figures 7A, B**). In another study, the researchers utilized Raman spectroscopy to analyze metabolic changes in spent embryo culture media (65). They observed significant metabolic signatures distinguishing embryos that progressed to the blastocyst stage from those that did not (**Figures 7C-E**). Despite challenges in clustering using PCA, a multilayer perceptron deep learning model achieved notable accuracy in distinguishing these groups, with a sensitivity of 77.78% and specificity of 72.00%. LDA identified key Raman shifts at 1008 cm^-1^, 1104 cm^-1^, and 1632 cm^-1^ as pivotal in distinguishing blastocyst from non-blastocyst embryos.

Raman spectroscopy has emerged as a valuable tool in assisted reproduction, facilitating non-invasive assessment of embryo quality and developmental potential. Scott et al. (66) applied Raman spectroscopy for evaluating embryo viability in IVF settings, reporting distinct implantation-related spectral patterns across day 3 and day 5 culture media. Their model achieved robust predictive performance (e.g., 82.4% sensitivity and 69% specificity on day 3, and perfect classification at a 0.43 threshold on day 5), underscoring potential of Raman as a non-invasive predictor of implantation success. Dos Santos ÉC et al. (67) expanded its utility by investigating metabolic profiles of *in vitro* bovine embryos, employing Raman spectroscopy to distinguish developmental kinetics. Spectral analysis across cleavage (22 hpc), post-embryonic genome activation (96 hpc), and blastocyst stages (168 hpc) identified distinctive profiles between fast and slow developing embryos (fast cleaved embryos *vs*. slow cleaved embryos at 22 hpc, fast embryos *vs*. slow embryos at 96 hpc, fast blastocysts *vs*. slow blastocysts at 168 hpc). This approach utilized a Triple T64000 Raman Spectrometer and included PCA and linear prediction to discern metabolic signatures such as lipids, DNA nitrogen bases, and proteins, contributing to differentiation among embryo groups based on developmental potential. The study underscored Raman spectroscopy’s role in selecting embryos with higher chances of successful pregnancy, thereby improving assisted reproduction outcomes. Further studies (68–70) reaffirmed Raman spectroscopy’s efficacy in assessing embryo viability through metabolomic analysis of embryo culture media. Seli’s (68) work demonstrated discriminatory spectral regions using genetic algorithms, with significant differences in viability indices between embryos that did and did not implant. Meng (69) integrated machine learning algorithms to predict pregnancy outcomes based on biochemical components identified by Raman spectroscopy, achieving 71.5% accuracy with convolutional neural network. Zhao (70) highlighted specific metabolites correlating with embryo viability, emphasizing Raman spectroscopy’s non-invasive potential to refine embryo selection processes in IVF treatments. In oocyte and embryo quality assessment, Ishigaki (71) and Okotrub et al. (72) utilized Raman spectroscopy to monitor molecular changes in lipid and protein concentrations during development and freezing, respectively. These studies revealed lipid transitions essential for cryopreservation and demonstrated Raman spectroscopy’s versatility in reproductive medicine.

Raman spectroscopy’s ability to provide real-time biochemical insights offers promising avenues for improving the selection and development of embryos in assisted reproduction. Its integration with advanced analytical techniques and machine learning enhances its utility in predicting reproductive outcomes and optimizing clinical strategies in IVF.

#### 2.3.2 Non-invasive genetic screening

**Figure 8:**
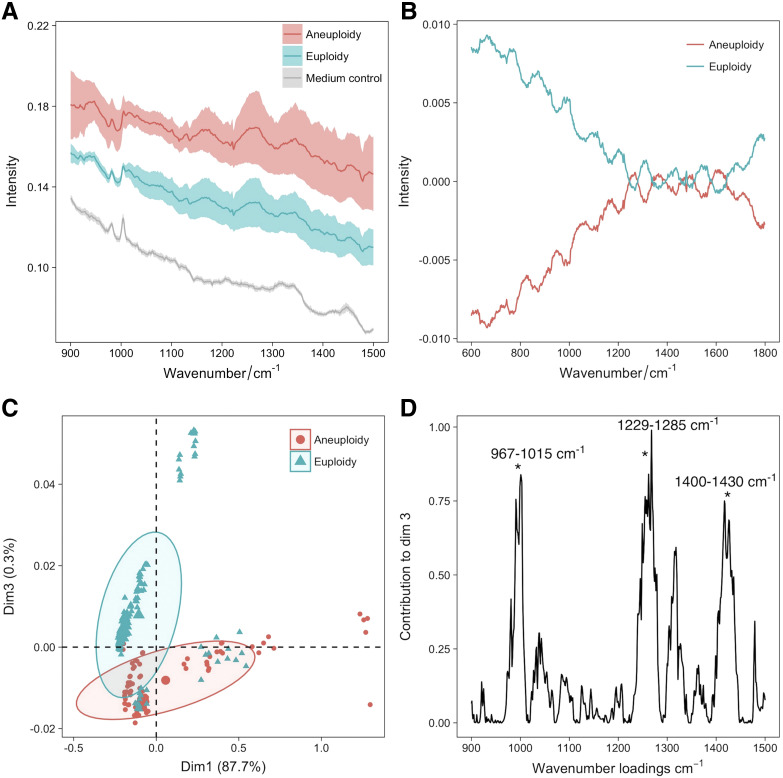
Raman spectroscopy for euploid and aneuploid oocytes classification. **(A)** averaged Raman spectrograms of spent embryo culture medium, showing differences between aneuploidy and euploidy; **(B)** mean-centered Raman spectrograms showing variations between aneuploidy and euploidy embryos; **(C)** PCA plot of Raman spectrograms showing clustering of neuploidy and euploidy embryos; **(D)** loading plots showing key Raman wavenumbers contributing to PCA dimension 3. Figures modified from Ref. (73).

Raman spectroscopy has limited but promising applications in non-invasive genetic screening Liang et al. (73) employed Raman spectroscopy combined with machine learning to detect chromosomal abnormalities in embryos. They analyzed 87 embryo culture medium samples (54 euploidy, 33 aneuploidy) and collected 220 euploidy spectra and 165 aneuploidy spectra. An additional validation set of 123 samples (61 euploidy, 62 aneuploidy) yielded 549 and 558 respective Raman spectra. For the reproductive potential assessment of human embryos, Raman spectroscopy was utilized to analyze spent embryo medium, revealing distinct metabolic differences between euploid and aneuploid embryos. Specific biomarkers (967–1015 cm^-1^, 1229–1295 cm^-1^, 1400–1430 cm^-1^) exhibited significant variations in metabolite concentrations, as demonstrated by Liang’s study (**Figure 8**). Machine learning models including k-nearest neighbors (kNN), random forests, and extreme gradient boosting achieved high accuracy (94.6% for kNN, 95.9% for stacking model) in classifying euploid and aneuploid embryos based on Raman spectra. This approach showcases the potential of Raman spectroscopy coupled with machine learning for non-invasive genetic screening and precise embryo selection in ART, with implications for improving IVF success rates and reducing pregnancy loss.

## 3 Perspectives

### 3.1 Advanced Raman techniques

The future of Raman spectroscopy in reproductive medicine holds tremendous potential, promising transformative advances in both diagnostics and therapeutic outcomes. As cutting-edge Raman techniques evolve, they are expected to deepen our understanding and management of reproductive health, particularly in ART. Methods such as SERS, CARS, SRS and LTRS each offer unique capabilities that make them particularly promising for future applications.

SERS, with its high sensitivity for detecting low-concentration biomarkers, could play a crucial role in analyzing the molecular composition of gametes and embryos, thereby identifying biomarkers associated with fertility and embryonic health (74). This development into non-invasive diagnostic tools may revolutionize early detection of reproductive disorders (75). CARS, known for its high signal intensity and spatial resolution (76), is well-suited for detailed, high-resolution imaging of gametes and embryos (77), enabling real-time observation of morphological and biochemical changes during fertilization and early embryonic development—insights that are essential for monitoring processes critical to successful reproductive outcomes (78, 79). Meanwhile, SRS, which offers enhanced sensitivity and faster imaging compared to traditional Raman techniques (80), is emerging as a powerful tool for real-time, label-free imaging of cellular processes (81, 82), making it highly promising for monitoring dynamic changes in gametes and embryos during ART (63). A notable extension of this approach is FSRS (83), which combines ultrafast temporal resolution with molecular specificity, enabling the capture of transient biochemical events during early embryo development. LTRS, which combines optical tweezers with Raman spectroscopy, provides a non-invasive approach to assessing embryo metabolic activity, thus contributing valuable data for embryo assessment and ART protocols (84). Future advancements, such as enhanced sensitivity, miniaturization of equipment, and sophisticated algorithms for signal analysis, will be vital in translating these technologies into clinical practice, ultimately improving reproductive outcomes.

### 3.2 Reproductive disorders related infertility

Raman spectroscopy is able to identify reproductive disorders like PCOS and endometriosis through biomarker detection, guiding personalized interventions for better patient outcomes (85). Huang et al. (59) employed Raman spectroscopy to analyze metabolic compositions in the follicular fluid of PCOS patients. They identified specific Raman biomarkers, which correlated with variations in oocyte developmental potential and clinical pregnancy rates. Raman spectroscopy has demonstrated significant efficacy in the assessment of oocyte quality and assumes a crucial position within the domain of clinical evaluations of reproductive health (86, 87). Endometriosis, characterized by the presence of endometrial tissue outside the uterus, often leads to infertility due to altered pelvic environment, impaired tubal function, ovarian dysfunction, immune alterations, and implantation failures. Laparoscopy, the gold standard for diagnosing endometriosis, is invasive and expensive, and cannot be used as a routine examination method. Ultrasound, another common diagnostic tool, is non-invasive but has limitations in sensitivity and specificity, often failing to detect small or deep lesions. Raman spectroscopy offers a non-invasive alternative with higher sensitivity and specificity, capable of early detection of molecular changes associated with endometriosis. This technique can analyze body fluids to identify biomarkers, providing rapid and accurate diagnosis, and aiding in early intervention and treatment (85). Raman spectroscopy proves invaluable in the specific detection and diagnosis of pelvic micro-endometriosis lesions that may evade detection by conventional ultrasound and serum CA125 examinations. Early identification of infertile women with combined pelvic microscopic and deep endometriosis lesions can inform optimal superovulation regimens, transplantation strategies, and luteal support regimens in IVF, ultimately improving pregnancy outcomes. Integrating Raman spectroscopy with other technologies, such as confocal microscopy and synchrotron radiation, further enhances its applications in ART, enabling high-resolution imaging and comprehensive molecular analysis of reproductive cells. Raman spectroscopy has transformative potential in revolutionizing ART practices and advancing reproductive medicine.

### 3.3 Cryopreservation techniques

Raman spectroscopy offers significant potential in the cryopreservation of embryos, oocytes, and sperm due to its ability to provide detailed molecular and biochemical information non-invasively and without labels. Current research demonstrates its applications in monitoring lipid phase transition, assessing mitochondrial activity, analyzing molecular structures, and evaluating the effects of cryoprotectants (**Table 1**). These capabilities are crucial for understanding and improving the integrity and functionality of cryopreserved cells (88). Future advancements in Raman spectroscopy, such as enhanced sensitivity, integration with other techniques, high-throughput screening, and clinical applications, could further optimize cryopreservation protocols and improve the success rates of ART like IVF and sperm banking. Thus, Raman spectroscopy holds great promise for advancing the field by enabling the development of more effective preservation strategies. The integration of Raman spectroscopy in cryopreservation research offers the potential for more consistent and successful reproductive outcomes in clinical settings, providing a sophisticated method to ensure the molecular integrity and viability of cryopreserved cells.

**Table 1:** Raman spectroscopy investigation of cryopreservation effects on mammal embryo, oocyte and sperm.

Objects	species	Indicators	Factors	Raman spectroscopy	Performances or biological significances	References
embryo	mouse	intracellular lipids	saturated stearic acid	Raman spectroscopy	saturated stearic acid exposure lowered intracellular lipids and unsaturation	T N Igonina et al., 2024 (93)
		fatty acid accumulation	deuterated stearic acid	Raman spectroscopy of isotopically labeled molecules	increases accumulation of deuterated stearic acid	A N Omelchenko et al., 2022 (94)
		lipid phase transition	linoleic acid	Raman spectroscopy	linoleic acid changes the biophysical characteristics of embryos’ lipidome, realized in lower lipid phase transition onset	T N Igonina et al., 2021 (95)
		photoresponse and redox state of cytochromes	/	Raman spectroscopy, cytochrome resonance Raman	abrupt changes in the electron transport chain work of frozen mouse embryos at temperatures below -50°C	E A Sazhina et al., 2019 (96)
		lipid phase transition	/	Raman spectroscopy	antisymmetric CH2 Raman peak, lipid phase transition occurs at the temperatures between -7 and 0°C	K A Okotrub et al., 2017 (97)
	cat	lipid phase transition	/	Raman spectroscopy	low lipid phase transition of lipid droplets provide a good background for successful application of slow freezing	Valentina I Mokrousova et al., 2020 (98)
oocyte & embryo	cat	lipid droplet phase transition	/	Raman spectroscopy	a promising tool for *in situ* monitoring of the lipid phase state in a single embryo/oocyte during its freezing	Konstantin A Okotrub et al., 2018 (72)
oocyte	cat	lipid phase transition	/	Raman spectroscopy of deuterium-labeled lipids	a promising tool for studying the lipid phase transitions and lipid redistributions inside single organelles within living cells	Konstantin A Okotrub et al., 2021 (99)
		lipid separation	/	Raman spectroscopy	evidence of lipid separation inside the lipid droplets in domestic cat oocytes during slow freezing	V I Mokrousova et al., 2020 (100)
	bovine	biochemical modifications of zona pellucida (ZP) and cytoplasm	/	Raman spectroscopy	biochemical modifications of ZP and cytoplasm, cold protein denaturation, oxidative damages	Giulia Rusciano et al., 2017 (62)
	mouse	survival rate, 5-methylcytosine (5-mC) expression, fertilization rate, two-cell rate, and blastocyst rate	L-proline	Raman spectroscopy	an appropriate concentration of L-proline can improve the cryopreservation efficiency of mouse oocytes	Lu Zhang et al., 2016 (101)
	ovine	cortical F-actin	/	Raman microspectroscopy	main differences: proteins (1657, 1440 and 1300 cm^-1^), F-actin cytoskeleton	Luisa Bogliolo et al., 2015 (61)
		modifications of ZP: ZP protein and carbohydrate components	/	Raman microspectroscopy	induce biochemical changes of ZP related to the secondary structure of proteins and carbohydrate residues: an increase in β-sheet content and a decrease in the α-helix content	Luisa Bogliolo et al., 2012 (60)
sperm	buffalo	mitochondrial activity, membrane integrity	/	near-infrared Raman	the damage induced by sperm sorting and freezing-thawing procedures can be quantified	Xiao-Xia Li et al., 2016 (21)
		mitochondrial activity, oxidative stress	melatonin	laser tweezers Raman spectroscopy	melatonin helps to protect buffalo sperm from reactive oxygen species	X X Li et al., 2012 (39)

### 3.4 Time-lapse imaging

Conducting longitudinal studies with time-lapse Raman imaging is crucial for understanding the dynamic biochemical changes that occur during gamete and embryo development. Time-lapse imaging involves capturing a series of images at set intervals over a period (89), allowing researchers to visualize and analyze the progression of events in real-time (90, 91). This methodology allows for continuous, non-invasive monitoring, providing real-time insights into molecular events critical for successful fertilization and early embryonic growth (92). By capturing fluctuations in molecular composition and metabolic activity, time-lapse Raman imaging reveals the health and viability of gametes and embryos over time, offering a comprehensive view of the temporal progression of biochemical processes and identifying deviations that may indicate developmental issues or compromised viability. This approach is instrumental in identifying critical biomarkers associated with successful fertilization and embryo development, thereby enhancing the efficacy of ART interventions. Specifically, identifying and validating Raman spectral markers for oocyte quality, sperm viability, and embryo health can significantly improve selection criteria in ART. The detailed molecular fingerprints obtained from Raman imaging reflect the biochemical composition and structural integrity of biological samples, allowing researchers to pinpoint biomarkers correlated with reproductive potential and developmental competence. Integrating these validated markers into clinical practice can enhance the selection process, enabling the identification of the most viable oocytes and embryos for fertilization and implantation. This targeted approach not only increases the likelihood of successful pregnancies but also reduces the physical and emotional burden on patients undergoing ART by optimizing procedural efficiency, thus advancing personalized and precise reproductive medicine.

### 3.5 Technical limitations and biological safety

Despite the promising applications of Raman spectroscopy in assisted reproduction, several critical limitations and challenges remain that must be addressed before its routine clinical adoption. To begin with, the inherently weak Raman signal and its susceptibility to background noise limit sensitivity, particularly when analyzing complex and heterogeneous biological samples such as sperm, oocytes, and embryos. Although advanced techniques like SERS and CARS have been developed to improve signal strength, their complexity and potential phototoxicity raise additional concerns. Moreover, the high cost of Raman instrumentation and the requirement for specialized expertise restrict its accessibility in clinical settings, as most applications are currently confined to research environments, with standardized protocols for clinical use still lacking. In addition, the interpretation of Raman spectra is highly dependent on advanced computational tools, often involving machine learning, yet there is no universally accepted set of spectral biomarkers or diagnostic criteria for gamete and embryo evaluation. This lack of standardization limits reproducibility across studies and hinders broader implementation. Furthermore, although Raman spectroscopy is widely regarded as non-invasive, its potential biological impact remains underexplored. Of particular concern is the possibility that laser exposure, even at low energy levels, may affect epigenetic stability during the critical windows of oocyte maturation and early embryonic development. Given the sensitivity of epigenetic imprinting during these stages, subtle perturbations could have downstream effects on developmental competence and offspring health. Therefore, addressing these limitations through continued technical refinement, clinical validation, and comprehensive safety assessments will be essential for the reliable integration of Raman spectroscopy into assisted reproductive technologies.

## 4 Conclusions

The applications of Raman spectroscopy in reproductive biology have profound implications for ART, with potential to significantly enhance diagnostics, treatment strategies, and patient outcomes. Raman spectroscopy provides precise molecular profiles for diagnosing male and female infertility, detecting biomarkers related to sperm quality and genetic integrity, and assessing oocytes and embryos for metabolic or structural deficiencies that affect conception. This non-invasive technique aids in selecting high-quality gametes and embryos for IVF procedures, improving implantation rates and reducing multiple pregnancies. The detailed molecular insights enable personalized treatment approaches, like antioxidant therapy for oxidative stress in sperm or nutrient supplementation for metabolic deficiencies in oocytes. Real-time monitoring of gametes and embryos during ART allows for early anomaly detection and optimization of culture conditions, improving embryo quality and implantation success. Raman spectroscopy also contributes to cryopreservation advancements by understanding biochemical changes during freezing and thawing processes, enhancing post-thaw survival rates. While its clinical value is increasingly evident, technical challenges such as low signal intensity, high equipment cost, and the need for standardization remain. Furthermore, concerns regarding potential biological effects, particularly during sensitive developmental windows, warrant continued investigation. With ongoing technological refinement and interdisciplinary collaboration, Raman spectroscopy is poised to become a transformative tool in the future of reproductive medicine.

## Glossary

ART: Assisted reproductive technology
CARS: Coherent anti-Stokes Raman scattering
SERS: Surface-enhanced Raman scattering
LTRS: Laser tweezers Raman spectroscopy
SRS: Stimulated Raman scattering
FSRS: Femtosecond stimulated Raman scattering
CCD: Charge-Coupled Device
IVF-ET: *In vitro* fertilization-embryo transfe
PCA: Principal component analysis
DFA: Discriminant function analysis
ICSI: IntracyIplasmic sperm injection
PGT-A: Preimplantation genetic testing for aneuploidies
NOA: Non-obstructive azoospermia
microTESE: Microdissection testicular sperm extraction
OA: Obstructive azoospermia
SCO: Sertoli cell-only
MA: Maturational arrest
IMM: Immature
MIV: *In vitro* matured
PC: Principal components
LDA: Linear discriminant analysis
PCOS: Polycystic ovary syndrome
ZP: Zona pellucida
IVF: *In vitro* fertilization
kNN: K-nearest neighbors
